# A noncanonical function of histidyl‐tRNA synthetase: inhibition of vascular hyperbranching during zebrafish development

**DOI:** 10.1002/2211-5463.12420

**Published:** 2018-04-17

**Authors:** Rui Ni, Lingfei Luo

**Affiliations:** ^1^ Key Laboratory of Freshwater Fish Reproduction and Development Ministry of Education Laboratory of Molecular Developmental Biology School of Life Sciences Southwest University Chongqing China

**Keywords:** angiogenesis, Cdh5, histidyl‐tRNA synthetase, Vegfa, zebrafish

## Abstract

Histidyl‐tRNA synthetase (Hars) catalyzes the ligation of histidine residues to cognate tRNA. Here, we demonstrate a noncanonical function of Hars in vascular development in zebrafish. We obtained a novel zebrafish *cq34* mutant which exhibited hyperbranching of cranial and intersegmental blood vessels 48 h after fertilization. The gene responsible for this phenotype was identified as *hars*. We found the increased expression of *cdh5* and *vegfa* in the *hars* ^*cq34*^ mutant. Knockdown of *cdh5* in the mutant reduced disordered connections of the hindbrain capillaries. Inhibition of vascular endothelial growth factor signaling suppressed the abnormal vascular branching observed in the mutant. Moreover, the human *HARS* mRNA rescued the vascular defects in the *cq34* mutant. Thus, the noncanonical function of Hars regulates vascular development, mainly by modulating expression of *cdh5* and *vegfa*.

**Abbreviations**
AARSs: aminoacyl‐tRNA synthetases
CCMs: cerebral cavernous malformations
Cdh5: vascular endothelial cadherin
CHX: cycloheximide
CtAs: central arteries
EC: endothelial cell
Hars: histidyl‐tRNA synthetase
hpf: hours postfertilization
HUVECs: human umbilical vein endothelial cells
ISVs: intersegmental vessels
Pdxl2: podocalyxin‐2
Tars: threonyl‐tRNA synthetase
Vegfa: vascular endothelial growth factor A

Vasculature is the earliest organ to form during vertebrate embryonic development. A functional vascular system is essential for continued embryonic development and adult survival. Thus, an aberrant vasculature contributes to the pathogenesis of numerous disease states, including cancer, ischemic diseases, infectious, and immune disorders 1, 2. Formation of the vascular system occurs through two fundamentally distinct processes: vasculogenesis and angiogenesis 3. Vasculogenesis defines the formation of a primitive vascular network. Angiogenesis refers to the formation of new blood vessels by sprouting of endothelial cells (ECs) from preexisting vessels and subsequent proliferation, migration, and remodeling. All of these processes are tightly regulated by a network of pro‐ and anti‐angiogenic factors.

During angiogenesis, ECs react to growth signals, allowing the sprouting cells to acquire a motile, invasive, and sprouting behavior by loosening contacts to adjacent cells and altering polarity. Newly formed blood vessels connect to the existing vasculature to establish a functional circulatory loop that permits the transportation of fluids, nutrients, circulating cells, hormones, and gasses. To attain such morphology, the correct polarity and spatial redistribution of EC–EC junctions must be achieved. Vascular endothelial cadherin (Cdh5) is the major component of endothelial adherens junctions, which is required for the organization of a stable vascular endothelium. The proper localization of Cdh5 is essential for establishing the vascular network. Loss of polarity will lead to disordered connections and multiple lumens. Under normal conditions, Cdh5 interacts and colocalizes with members of the Par polarity complex to regulate the accumulation and redistribution of podocalyxin‐2 (Pdxl2) at the apical surface, Pdxl2 then recruits other apical proteins as the polarity and vascular lumens begins to form 4. Altered Cdh5 localization may cause incorrect EC polarity and contribute to diseases, including so‐called cerebral cavernous malformations (CCMs) and tumors.

The mechanisms and pathways involved in vascular development are highly conserved among vertebrates. The vascular endothelial growth factor (VEGF) is the major contributor to regulating vessels’ growth 5. The pro‐angiogenic ligand vascular endothelial growth factor A (VEGFA), secreted by a variety of cell types, is the master regulator as it binds to the VEGFR2 receptor on angioblasts and new vessels, from where it activates downstream signaling and mediates several processes in ECs. Mice lacking functional VEGF signaling will present various developmental defects, such as fewer ECs, and will fail to form a functional vasculature 6, 7. Similar phenotypes have been reported in *Xenopus* and chick 8, as well as zebrafish 9. VEGF has been reported to induce phosphorylation of tyrosine 685 on CDH5 in a reaction mediated by Src tyrosine kinase. This process is critical for VEGF‐induced EC migration.

Aminoacyl‐tRNA synthetases (AARSs) catalyze the ligation of amino acids to their cognate tRNA, in what constitutes the first step of translation. However, recent studies indicate that some AARSs possess noncanonical functions in homeostatic processes 10, including the regulation of angiogenesis. Tryptophanyl‐tRNA synthetase and tyrosyl‐tRNA synthetase directly regulate angiogenesis as anti‐ and pro‐angiogenic cytokines 11. Glutamyl‐prolyl‐tRNA synthetase negatively regulates angiogenesis by blocking VEGFA translation 12. In addition to the above *in vitro* studies*, in vivo* experiments using zebrafish mutants in seryl‐tRNA synthetase, isoleucyl‐tRNA synthetase, and threonyl‐tRNA synthetase (*tars*) exhibiting abnormal vascular sprouting suggested interesting and surprising roles for the different synthetases 13, 14, 15, 16. The discovery of noncanonical functions of AARSs in vertebrates improves our understanding of vascular development and AARSs. It is not clear whether the other AARSs play any role in regulating angiogenesis *in vivo*.

In this study, we obtained a novel zebrafish *cq34* mutant with increased branching angiogenesis. By analyzing the mutants, we identified that these phenotypes are caused by a mutation in the post‐transcriptional splice recognition site at the histidyl‐tRNA synthetase (*hars*) genomic locus, which resulted in the deletion of exon 7. We demonstrate that Hars possesses a noncanonical function responsible for inhibiting vascular hyperbranching during zebrafish development. Deficiencies in Hars function result in the increased expression of *cdh5* and *vegfa*. Injection of human *HARS* mRNA rescued the vascular defects in the *cq34* mutant, indicating the noncanonical function of Hars is conserved between zebrafish and humans. Our results suggest that the noncanonical function of Hars in the regulation of vascular development mainly depends on the modulating the expression of *cdh5* and *vegfa*.

## Materials and methods

### Zebrafish strains

Zebrafish (*Danio rerio*) of the AB genetic background, *Tg(kdrl:GFP)* transgenic line, and *cq34* mutant were maintained under standard laboratory conditions according to institutional animal care and use committee protocols. Embryos were treated with 0.003% 1‐phenyl‐2‐thiourea (PTU; Sigma, St. Louis, MO, USA) from 24 hours postfertilization (hpf) to inhibit pigmentation.

### Genetic mapping

The *cq34* locus was defined by genotyping of *cq34* mutant embryos using simple sequence length polymorphic (SSLP) markers on the linkage group 14 (LG 14). We finally identified the mutation in zebrafish *hars* genomic DNA at the first base pair of intron 7–8 (substitution from G to A), resulting in the deletion of exon 7 which contains 99 base pairs coding amino acid from 211 to 243. To perform genotyping of the *cq34* mutation, we amplified the *cq34* locus from the isolated genomic DNA and cDNA by PCR using the following primers, the *hars* primers for genomic DNA: 5′‐AATCAAATCTCAAGTCTCTTCAT‐3′ and 5′‐TTTACTCACCCTGCATGCTG‐3′; the *hars* primers for cDNA: 5′‐GGGCCGATACAGGGAGTTTT‐3′ and 5′‐GCGCCAGACTCAGATCAAAC‐3′. The *cq34* mutation was determined by direct sequencing of the amplified fragments.

### Morpholino and mRNA injections

Antisense morpholino oligonucleotides against *cdh5* (*cdh5MO*: 5′ ‐TTTACAAGACCGTCTACCTTTCCAA‐3′) were obtained from GeneTools (Philomath, OR, USA) and injected into the yolk at 1‐ to 4‐cell stage embryos. Total RNA was extracted using Trizol (Life Technologies, Carlsbad, CA, USA) and reverse transcribed to cDNA using Omniscript reverse transcriptase kit (QIAGEN, Stockach, Germany). Amplified zebrafish *hars* and human *HARS* cds fragment and subcloned into the PCS2+. Capped mRNA were synthesized from the linearized PCS2+ constructs using the mMESSAGE mMACHINE (Ambion, Carlsbad, CA, USA). Synthetic mRNA were injected into the yolk at 1‐ to 2‐cell stage embryos.

### Whole‐mount *in situ* hybridization and quantitative real‐time PCR

*In situ* hybridization and quantitative real‐time PCR were performed as previously described 17, 18. The primers for synthetic probes were as follow: *hars* primers, 5′‐CGCAGTATCTGCAGTTCGGT‐3′ and 5′‐TCCCTGATTCGATCGGCTAC‐3′; *vegfa* primers, 5′‐ACTCACCGCAACACTCCACT‐3′ and 5′‐GAGCAAGGCTCACAGTGGTT‐3′; *cdh5* primers, 5′‐GGGCTGTTGAAGAGAACCGA‐3′ and 5′‐ GGGCACTGACACTCATTCCA‐3′.

### Treatment with chemicals

The embryos were treated with either 1 μm SU5416 (Sigma) or 50 μm cycloheximide (CHX; Sigma) from 36 hpf to observation, or treated with DMSO at the same concentration as control.

### Immunofluorescence staining and imaging

Human umbilical vein endothelial cells (HUVECs, ALLCELLS) were cultured according to standard protocol. Cell immunofluorescence staining was performed as previously described 19 with anti‐HARS antibodies (1 : 100; Abcam, ab71305, Cambridge, UK). Anti‐zebrafish‐Cdh5 antibodies (1 : 200, a gift from Markus Affolter) and anti‐zebrafish‐Pdxl2 antibodies (1 : 200, a gift from Markus Affolter) 20 were visualized using donkey anti‐rabbit AlexaFluor 647 (Invitrogen, Carlsbad, CA, USA). Anti‐GFP antibodies (1 : 400; Santa Cruz Biotechnologies, Santa Cruz, CA, USA) were visualized using goat anti‐mouse AlexaFluor 488 (Invitrogen). The fluorescence micrographs were imaged using a LSM780 confocal microscope equipped with ZEN2010 software (Carl Zeiss, Oberkochen, Germany). Bright views and *in situ* hybridization images were captured using a SteREO Discovery 20 microscope equipped with axiovision rel 4.8.2 software (Carl Zeiss). The imaging was performed as previously described 21, 22.

### Western blotting

For the western blotting, we constructed the *hars(wt)‐eGFP* and *hars(mutant)‐eGFP* in *hsp* plasmids. The plasmids were microinjected into zebrafish. We heat shock the embryos at 38.5 °C at 36 hpf, and embryos were harvested and lysed at 50 hpf. The western blotting was performed using anti‐GFP antibodies (1 : 4000; abcam) and anti‐a‐Tubulin (Sigma) antibodies. All the experimental procedures were performed as previously described 17.

### Hars activity assay

The embryonic lysates (50 embryos at 72 hpf in a tube) were prepared using the HARS ELISA Kit (JL45833; Shanghai Jianglai Biotech, Shanghai, China) which is suited for detecting zebrafish Hars activity, according to the manufacturer's instruction. The zebrafish Hars activity was measured at a wavelength of 450 nm in the SPECTRA MAX 190 (Molecular Devices 22).

## Results

### The *cq34* mutants display hyperbranching cranial and intersegmental blood vessels

**Figure 1:**
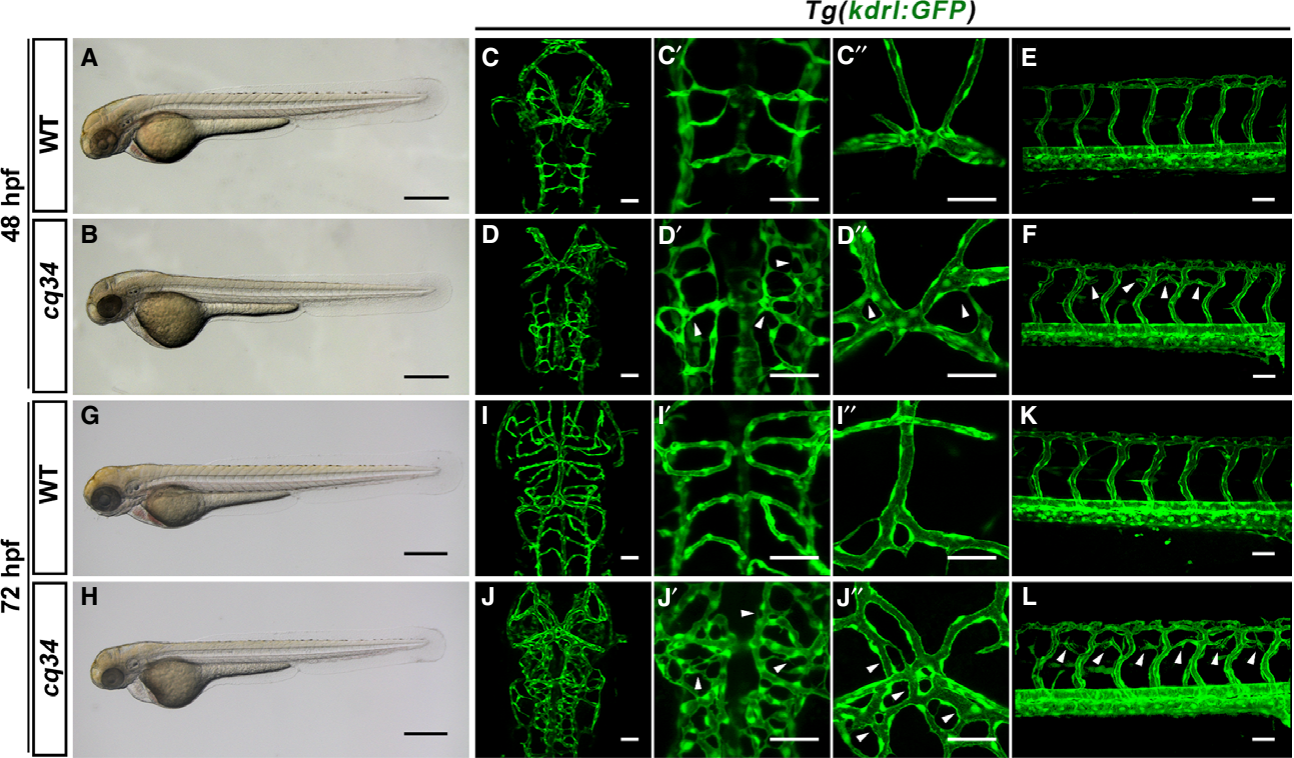
The *cq34* mutants exhibit hyperbranching cranial and intersegmental blood vessels. (A,B,G,H) Bright‐filed micrographs of the *cq34* mutant reveal a decreased head and thin trunk at 48 hpf, and a pericardial edema at 72 hpf. (C–F, I–L) Blood vessels are visualized using the *Tg(kdrl:GFP)* transgenic line. The *cq34* mutants display ectopic branching (arrowheads) in the head and dorsal part of the trunk at 48 hpf, which become more severe at 72 hpf. The aberrant branching of CtAs (C′, D′, I′, J′) and dorsal cranial vasculature (C″, D″, I″, J″) can be observed clearly under higher magnification. Scale bars, 100 μm (A,B,G,H), 50 μm (C–F, I–L).

Identification of new vascular regulators enables us to better understand the cellular and molecular mechanisms of vascular network formation. To isolate new vascular regulators, we performed an *N*‐ethyl *N*‐nitrosourea (ENU) mutagenesis screening. We identified an embryonic lethal mutant, *cq34*, which presented vascular defects after 48 hpf. In bright‐field microscopy, the *cq34* mutants could be distinguished from wild‐type (WT) individuals by the slightly decreased size of the head, eyes, and overall body at 48 hpf (Fig. 1A,B). Using the *Tg(kdrl:GFP)* line, the *cq34* mutants displayed increased sprouting of angiogenic vessels in the head and ectopic intersegmental vessels (ISVs) branching in the dorsal part of the trunk at 48 hpf (Fig. 1C–F). At 72 hpf, a pericardial edema was observed in mutants (Fig. 1G,H). At the same time, the abnormal branching of central arteries (CtAs) and cranial vessels became increasingly chaotic, and the aberrant angiogenic sprouting emanating from the ISVs became even more severe in the *cq34* mutants (Fig. 1I–L). The mutants did not exhibit any apparent morphologic phenotype during early embryogenesis, vasculogenesis proceeded normally, and circulatory flow could be detected during neovascularization. These results indicate that the gene affected by the *cq34* mutation acts as a regulator of angiogenesis.

### The gene responsible for the *cq34* encodes zebrafish Hars

**Figure 2:**
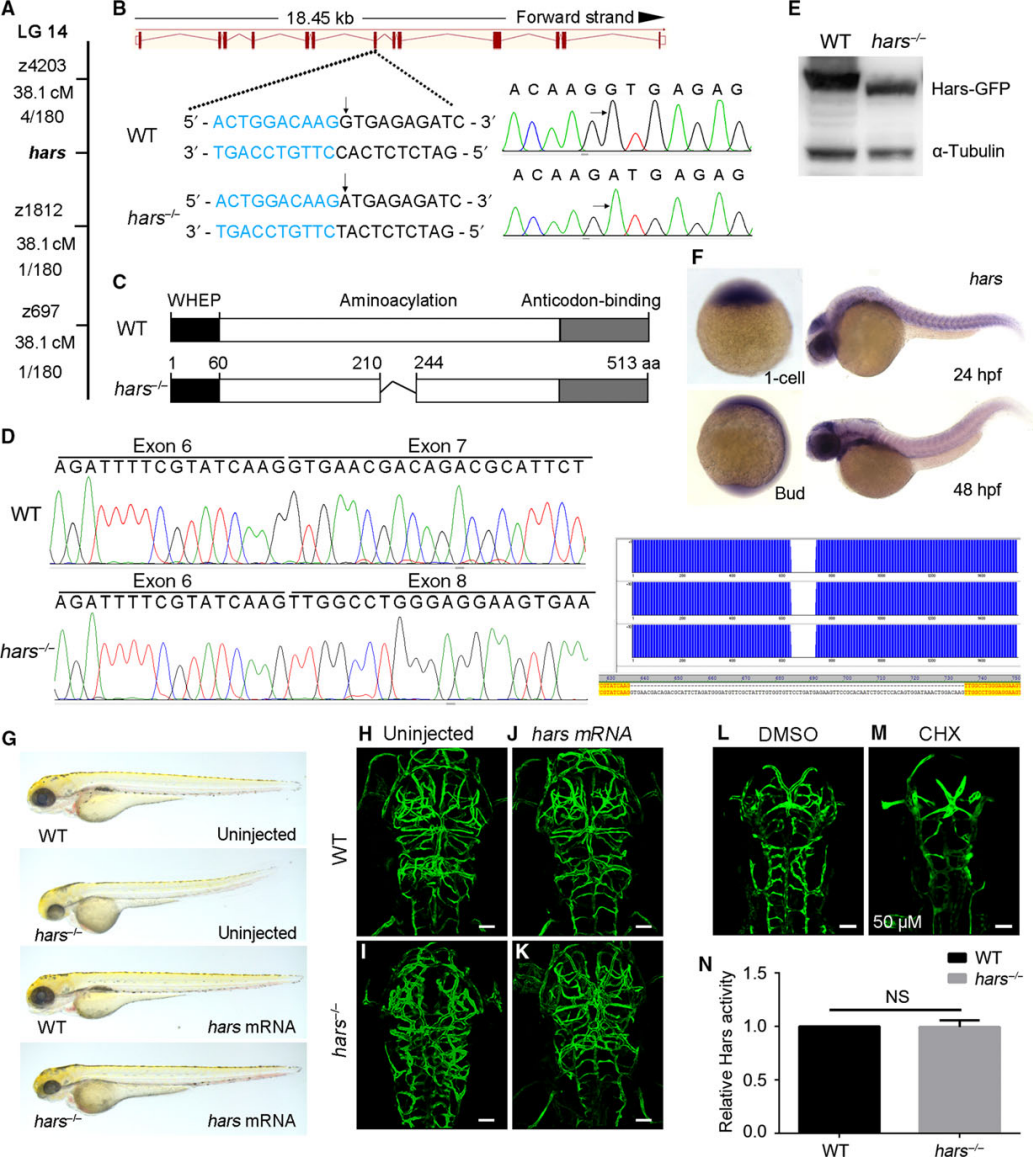
The gene responsible for the *cq34* encodes Hars. (A) The *cq34* mutation is located on the LG 14. Numbers below the SSLP markers indicate the genetic distance and the recombination events seen in 180 mutant embryos. (B) The *hars* genomic DNA sequence of WT and *hars* ^*−/−*^ shows a point mutation in the first base pair of intron 7–8 (substitution from G to A), causing deletion of exon 7. Exon bases are indicated in blue and intron bases in black. (C) Schematic representation of the secondary structure of Hars, which consists of WHEP, aminoacylation, and anticodon‐binding domains. The *hars* ^*−/−*^ mutant presents a partial deletion of the aminoacylation domain (amino acids: 211–243). (D) Sequencing the *hars* cDNA of WT and *hars* ^*−/−*^ shows the exon 7 deletion in mRNA level in *hars* ^*−/−*^. (E) Western blotting shows the deletion in protein level in *hars* ^*−/−*^, and α‐Tubulin serves as a loading control. (F) Whole‐mount *in situ* hybridization shows that the *hars* transcript is expressed in one‐cell stage embryos and thus is maternally supplied. It is expressed ubiquitously in the embryo at later stages. (G–K) Injection of WT *hars* mRNA prevents the appearance of body defects and ectopic brain vascular branching typical of the *cq34* mutant at 72 hpf. (L,M) Inhibition of protein synthesis by CHX treatment between 36 and 48 hpf leads to reduced and thinner brain vasculature, and DMSO treatment was used as control. (N) *In vivo* Hars activity measured by ELISA assay. *n* = 3 tubes of lysates, mean ± SD, NS, not significant, Student's *t*‐test. Scale bars, 50 μm.

Genome mapping and positional cloning were performed to identify the defective locus in the *cq34* mutant. The *cq34* mutant was mapped to the *hars* gene on LG 14 (Fig. 2A) and was further characterized as having a G to A mutation in the first base pair of intron 7–8 (Fig. 2B). This represents an important post‐transcriptional splice recognition site and results in the deletion of exon 7, which contains 99 base pairs (Fig. 2D). *Hars* is one of the 20 AARSs that catalyze the ligation of amino acids to cognate tRNA. The *cq34* mutant (*hars* ^*−/−*^) presented a partial deletion of the aminoacylation domain (amino acids: 211–243; Fig. 2C). We performed the western blotting to show the deletion in protein level (Fig. 2E).

We performed a rescue experiment to confirm that *hars* was indeed the *cq34* gene. Accordingly, we injected WT *hars* mRNA into the yolk at one‐ to two‐cell stage embryos and found that the injection effectively prevented the body defects and hyperbranching brain vasculature observed in the mutants at 72 hpf (Fig. 2G–K). This finding confirms that the disorganization of vascular patterning in the mutants is due to the loss of zygotic Hars function.

We examined the spatiotemporal pattern of *hars* expression during development. Whole‐mount *in situ* hybridization showed that *hars* transcripts were strongly expressed in one‐cell stage embryos, indicating that *hars* is a maternal gene. Maternally supplied mRNA and protein initially compensate for the lack of zygotic Hars function, which may explain the lack of early phenotypes in *hars* ^*−/−*^. *Hars* was expressed in the bud stage and particularly in cranial tissues and trunk somites adjacent to sites of vessel growth at 24 hpf. After that, it was expressed ubiquitously in the embryo (Fig. 2F).

*Hars* encodes an enzyme that is required for protein synthesis. To investigate whether the canonical function of Hars is needed for increased vascular sprouting, we inhibited the general protein synthesis following treatment with CHX. The intervention decreased brain vasculature (Fig 2L,M), but caused no aberrant vascular sprouting. In addition, the mutation does not affect the aminoacylation activity (Fig. 2N). These suggest that a noncanonical function of Hars, instead of its canonical function, is involved in vascular development.

### The *hars* mutants display disordered connections

**Figure 3:**
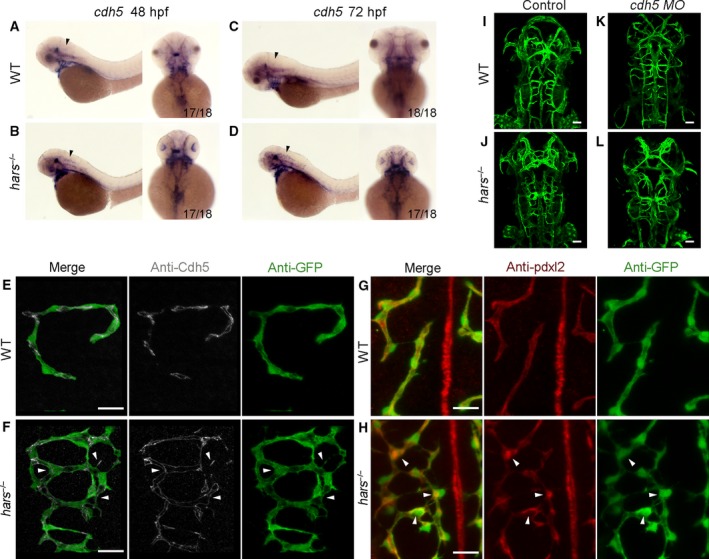
The *hars* mutants display disordered connections. (A–D) *In situ* hybridization shows that *cdh5* expression is upregulated in the brain of *hars* ^−/−^ at 48 and 72 hpf (arrowheads). (E,F) Antibody staining shows the Cdh5 distribution overlapping with the abnormal branching connections of CtAs, and additionally, Cdh5 appears as punctate clusters at some sprouting sites (arrowheads) in *hars* ^*−/−*^ at 48 hpf. (G,H) Pdxl2 is seen to accumulate at sprouting and connection disordered sites (arrowheads) in *hars* ^*−/−*^ at 48 hpf. (I–L) Injection of 2.5 ng *cdh5 MO* reduces the abnormal brain vascular connections in *hars* ^*−/−*^ at 48 hpf, but the vasculature becomes immature. Scale bars, 20 μm (A,B,G,H), 50 μm (I–L).

An obvious disorganization of CtAs connections was evident in the mutants. Previous studies have indicated that Cdh5 is responsible for endothelial adherens junction organization 23, 24 and can affect vascular patterning by regulating EC polarity. *In situ* hybridization showed significantly increased *cdh5* expression in cranial tissues of the mutants at 48 and 72 hpf (Fig. 3A–D). The mutant presented vascular hyperbranching with more disordered connections and mature lumens in the brain vasculature. We found that Cdh5 decorated cell–cell junctions showed strong overlap with the vascular network. At the same time, Cdh5 distribution was substantially altered, with Cdh5 appearing in punctate clusters at some sprouting sites in *hars* ^*−/−*^ at 48 hpf (Fig. 3E,F). These results show that the mutant exhibited disordered connections and strong cell junctions. We further examined the localization of the polarity‐related protein Pdxl2, which is normally found on the apical side of ECs and can be regulated by Cdh5. Formation of the lumen is initiated by the polarization of cell membranes. We observed increased Pdxl2 accumulation at the sprouting and connection disorder sites in *hars* ^*−/−*^ at 48 hpf (Fig. 3G,H). This finding indicates inappropriate EC polarity in the mutant. Knockdown of *cdh5* by injection of morpholino reduced the number of disordered CtAs connections in *hars* ^*−/−*^ at 48 hpf, but the vasculature became immature (Fig. 3I–L). These data suggest that Hars influences *cdh5* expression. Cdh5 is an important structural component of the endothelial and is also involved in the formation of vascular network.

### The noncanonical function of Hars influences *vegfa* expression

**Figure 4:**
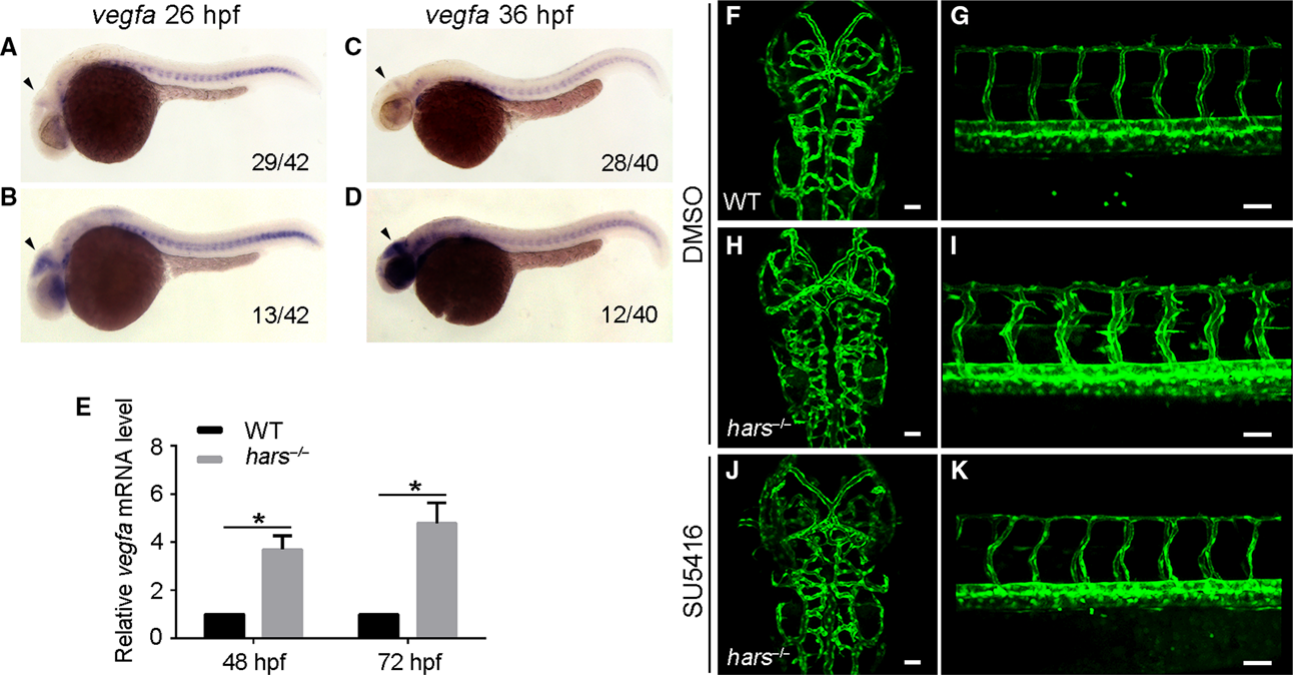
Noncanonical function of Hars represses *vegfa* expression. (A–D) *In situ* hybridization reveals that the expression of *vegfa* in the head is upregulated in about 25% of embryos at 26 and 36 hpf (arrowheads). (E) Quantitative real‐time PCR shows that *vegfa* mRNA levels in *hars* ^*−/−*^ are significantly upregulated at 48 and 72 hpf. Target transcript levels were normalized to the reference gene *elf1a* and to levels in WT controls, which were set to 1; *n* = 3 pooled biological replicates of ≥ 20 embryos, with three technical replicates per *n*. (mean ± standard error of the mean, *P < 0.05, two‐tailed *t*‐test). (F–K) SU5416 treatment suppresses ectopic branching in the brain and trunk of *hars* ^*−/−*^ at 60 hpf. Scale bars, 50 μm.

Secreted Vegfa is the principal master regulator of new blood vessel sprouting 25. We observed strong neovascularization in the *hars* mutant. To determine the relevance of *vegfa* for neovascularization in *hars* ^*−/−*^, we examined its expression. *In situ* hybridization showed that the expression of *vegfa* was obviously upregulated in the cranial tissues in about 25% of the embryos at 26 and 36 hpf (Fig. 4A–D). Embryos with increased *vegfa* expression were identified as mutants by sequencing. Quantitative real‐time PCR was used to assay the expression levels of Vegf ligands at 48 and 72 hpf, when abnormal branching was observed. Only the expression of *vegfa* was significantly upregulated at 48 hpf (3.69‐fold) and 72 hpf (4.79‐fold) in the mutants (Fig. 4E). This indicates that Hars influences *vegfa* expression.

We treated embryos with the Vegf receptor inhibitor SU5416 at 36 hpf, when the abnormal vasculature began to appear. Confocal analysis of the brain and trunk vasculature at 60 hpf showed that the ectopic CtAs and ISVs sprouting in *hars* ^*−/−*^ were completely blocked by SU5416 treatment (Fig. 4F–K). These data indicate that the noncanonical function of Hars negatively regulates *vegfa* expression, which normally stimulates angiogenesis.

### The role of Hars in regulating vascular development is functionally conserved between zebrafish and humans

**Figure 5:**
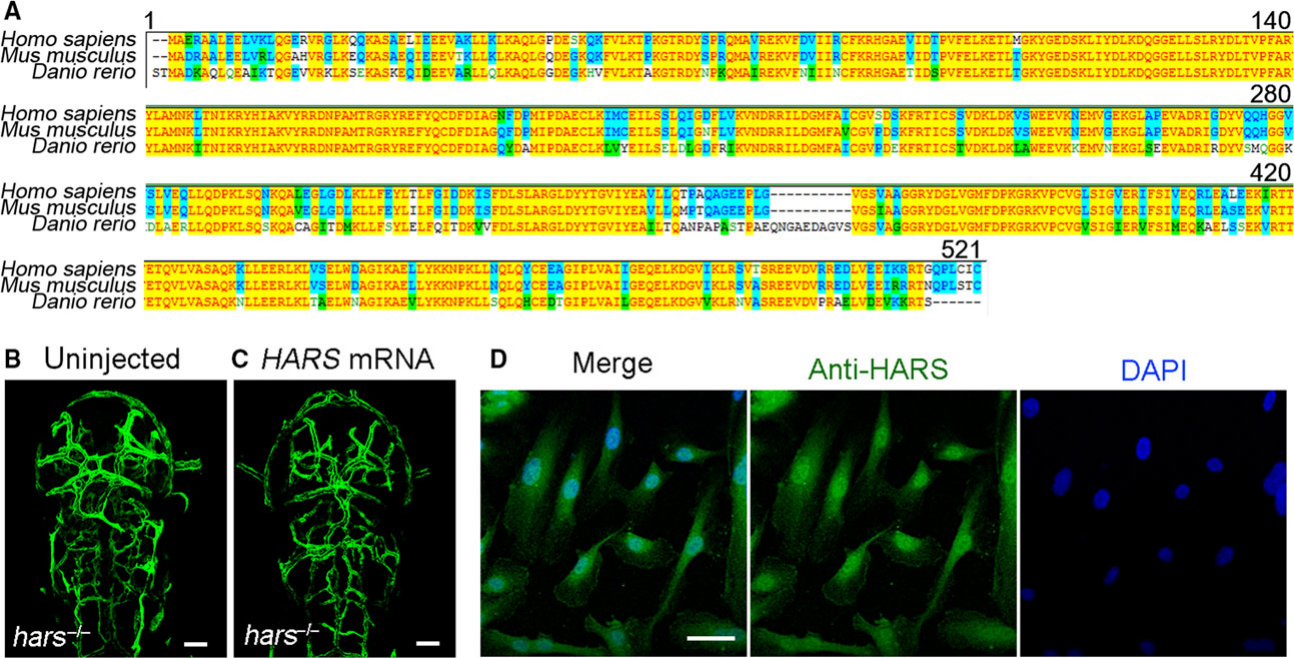
The role of Hars in regulating vascular development is functionally conserved between zebrafish and humans. (A) Hars protein sequence alignment shows high homology between the three species tested. (B,C) Injection of human *HARS* mRNA rescues ectopic brain vascular branching in zebrafish *hars* ^*−/−*^, indicating a conserved regulatory function of Hars in vascular development between zebrafish and humans. (D) Confocal immunofluorescence microscopy shows nuclear localization of endogenous HARS in HUVECs. Scale bars, 50 μm.

Alignment of Hars protein sequences from three species showed that zebrafish and mammalian HARS were highly homologous (Fig. 5A). Furthermore, injection of human *HARS* mRNA effectively abolished ectopic CtAs branching in the zebrafish mutants (Fig. 5B,C). This indicates the remarkable functional conservation of Hars between zebrafish and humans. It is generally believed that HARS is localized in the cytoplasm, where it participates in protein synthesis. However, confocal immunofluorescence microscopy demonstrated the nuclear localization of endogenous HARS in HUVECs (Fig. 5D), indicating a possible novel nuclear function for Hars.

## Discussion

Zebrafish have emerged as an exceptional model for studying vascular development 26. In the present study, we demonstrate, for the first time, that Hars possesses a noncanonical function in vertebrates. This noncanonical function was confirmed by identifying a zebrafish mutant with increased energetic filopodia and a chaotic vascular network at 48 hpf. The finding suggested that loss of Hars noncanonical function stimulated angiogenesis.

Cdh5 is located at cell‐to‐cell contact points, where it mediates adhesion and transfer of intracellular signals. We found increased *cdh5* expression and altered Cdh5 localization in *hars* ^*−/−*^. Given that, as a junction component, Cdh5 plays role in ECs connection and lumen formation, knockdown of *cdh5* reduced the disordered connections, but vasculature became thinner in the mutant. This suggests that Cdh5 is involved in creating or maintaining cell‐to‐cell connection function, leading to disordered connections in the mutants. Previous studies have reported that *cdh5* can regulate the polarization of ECs. In some cases, defects in EC polarity may cause pathologies, such as CCMs. Abnormal polarity was observed in *hars* mutant. In addition, brain vascular defects, especially in the cranial vessels and CtAs of the *hars* mutants, were similar to the cerebrovascular disorders in CCMs. These findings indicate that Hars may be associated with CCMs and may provide useful information for further research on possible therapies against CCMs diseases.

In this study, we showed increased *vegfa* expression in *hars* ^*−/−*^. Moreover, the vascular defects observed in the mutant were blocked upon treatment with a Vegf receptor inhibitor, indicating that the function of Hars in regulating vascular sprouting depended on Vegfa signaling during vascular development. Thus, Hars may be a promising pharmacological target in neovascular diseases and cancers.

Both Cdh5 and Vegf signaling pathways are required for angiogenesis, and they play roles in regulating vascular patterning in *hars* ^*−/−*^. *Cdh5* regulates the connections, and *vegfa* stimulates vascular sprouting and growth. We speculate that the noncanonical function of Hars negatively regulates the expression of *cdh5* and *vegfa*, thereby inhibiting vascular hyperbranching during zebrafish development.

Recent studies have documented a variety of important noncanonical functions for AARSs. Furthermore, their specific pro‐ and anti‐angiogenic activities have been attributed to the N‐ or C‐terminal accessory domains of tRNA synthetases *in vitro* 27, 28, 29, 30, 31, 32. Although the mutation site of *hars* ^*−/−*^ is not located in the WHEP, but rather in the aminoacylation domain, it is likely to undermine the structural integrity of the WHEP domain. This may prevent Hars from binding to other proteins or promoters. Although the *tars* zebrafish mutant displays ectopic branching of cranial and trunk vessels, similar to that shown here in *hars* ^*−/−*^, WT *tars* mRNA could not reduce the vascular defects in *hars* ^*−/−*^. These findings indicate that multiple complex mechanisms mediate the linkage between AARSs and angiogenesis separately, and each AARS plays an irreplaceable role in regulating angiogenesis. Given that some of the HARS protein is distributed in the nucleus, HARS may be a new transcriptional factor that binds directly to target promoters.

Having shown that the angiogenesis‐regulating role of Hars is conserved between zebrafish and humans, we speculate that impairment of human HARS may be associated with vascular disease. Thus, zebrafish *hars* ^*−/−*^ may present a unique and clinically relevant angiogenesis model. Our data uncovering this new noncanonical function of Hars will pave the way for further insights into regulatory pathways controlling angiogenesis.

## Author contributions

LL and RN designed the experimental strategy, analyzed data and wrote the manuscript. RN performed all the experiments.
